# Draft Genome Sequence of the Probiotic *Bifidobacterium longum* subsp. *longum* Strain MC-42

**DOI:** 10.1128/genomeA.01411-16

**Published:** 2016-12-15

**Authors:** Alexey E. Tupikin, Anna I. Kalmykova, Marsel R. Kabilov

**Affiliations:** a SB RAS Genomics Core Facility, Institute of Chemical Biology and Fundamental Medicine, Siberian Branch of the Russian Academy of Sciences, Novosibirsk, Russia; b Closed Joint-Stock Company “Biovesta,” Novosibirsk, Russia

## Abstract

Here, we report the draft genome sequence of *Bifidobacterium longum* subsp. *longum* strain MC-42 isolated from the feces of a healthy infant, and which was used in the commercially available probiotic product Biovestin.

## GENOME ANNOUNCEMENT

Bifidobacteria, belonging to the *Actinobacteria* group, are Gram-positive bacteria with high G+C content that are commonly found in the human gastrointestinal tract (1). Members of genus *Bifidobacterium* are usually used as probiotic organisms, which provide a health benefit to the host (2). *Bifidobacterium longum* is widely used as a probiotic due to its resistance to the high acidic environment during the gastric transit (3, 4). Here, we present the draft genome sequence of *B. longum* subsp. *longum* strain MC-42 isolated from the feces of a healthy infant, and which was used in the commercially available probiotic product Biovestin.

Genomic DNA of *B. longum* was isolated from cultured cells by using a GeneJET genomic DNA purification kit (Thermo Scientific) according to the manufacturer's instructions. Then, genomic DNA was sheared in a microTUBE AFA fiber snap-cap tube using a Covaris S2 instrument (Covaris) with a medium size distribution of fragments of about 500 bp. The paired-end library was prepared using a NEBNext Ultra DNA library prep kit for Illumina (NEB). Whole-genome sequencing of the *B. longum* library was conducted on a MiSeq genome sequencer (2 × 300 cycles, Illumina) in the SB RAS Genomics Core Facility (ICBFM SB RAS, Novosibirsk, Russia). The entire genome was assembled *de novo* with CLC Genome Workbench software v8.5 (CLC Bio). The automatic functional annotation results were obtained using the NCBI Prokaryotic Genome Annotation Pipeline (PGAAP) (http://www.ncbi.nlm.nih.gov/genome/annotation_prok/).

A total of 924,022 reads were generated from the *B. longum* genome. Reads were assembled to a draft genome of 2,287,827 nucleotides at 80-fold coverage with 59.8% G+C content. The genome sequence consists of 29 contigs. The *B. longum* MC-42 genome contains 1,827 coding sequences, four rRNA operons, and 51 tRNA genes. A total of 46 pseudogenes, three noncoding RNA (ncRNA) genes, two clusters of regularly interspaced short palindromic repeat (CRISPR) systems, and 22 frameshifted genes were predicted using PGAAP.

### Accession number(s).

The whole-genome shotgun project of *B. longum* has been deposited in GenBank under the accession number LNCM00000000.
